# Treatment-dependent and treatment-independent risk factors associated with the risk of diabetes-related events: a retrospective analysis based on 229,042 patients with type 2 diabetes mellitus

**DOI:** 10.1186/s12933-015-0179-2

**Published:** 2015-02-03

**Authors:** Thomas Wilke, Sabrina Mueller, Antje Groth, Andreas Fuchs, Lisa Seitz, Joachim Kienhöfer, Ulf Maywald, Rainer Lundershausen, Martin Wehling

**Affiliations:** IPAM, University of Wismar, Philipp-Müller-Str. 12, 23966 Wismar, Germany; AOK PLUS, Sternplatz 7, 01067 Dresden, Germany; Novo Nordisk Pharma GmbH, Brucknerstraße 1, 55127 Mainz, Germany; Diabetes Centre Erfurt/Bad Berka, Thälmannstraße 25, 99085 Erfurt, Germany; Clinical Pharmacology, Mannheim/Center for Gerontopharmacology, Medical Faculty Mannheim, University of Heidelberg, Maybachstr. 14, 68169, Mannheim, Germany

**Keywords:** Type 2 diabetes mellitus, Diabetes-related events, Macrovascular event risk, Mortality risk of type 2 diabetes mellitus patients, HbA_1C_

## Abstract

**Background:**

The aim of this study was to analyse which factors predict the real-world macro-/microvascular event, hospitalisation and death risk in patients with type 2 diabetes mellitus. Furthermore, we aimed to investigate whether there exists both an under- and over-treatment risk of these patients.

**Methods:**

We used a German claims/clinical data set covering the years 2010–12. Diabetes-related events were defined as (1) macro-, (2) microvascular events leading to inpatient hospitalisation, (3) other hospitalisations with type 2 diabetes mellitus as main diagnosis, (4) all-cause death and (5) a composite outcome including all event categories 1–4. Factors associated with event risk were analysed by a Kaplan-Meier curve analysis and by multivariable Cox regression models.

**Results:**

229,042 patients with type 2 diabetes mellitus (mean age 70.2 years; mean CCI 6.03) were included. Among factors that increased the event risk were patients’ age, male gender, the adapted Charlson Comorbidity Index, the adapted Diabetes Complication Severity Index, previous events, and number of prescribed chronic medications. For systolic blood pressure/HbA_1C_, a double-J/U-curve pattern was detected: HbA_1C_ of 6–6.5% (42-48 mmol/mol) and systolic blood pressure of 130-140 mmHg (17.3-18.7kPa) were associated with the lowest event risk, values below/above that range were associated with higher risk. However, this pattern was mainly driven by the death risk and was much less clearly observed for the macrovascular/microvascular/hospitalization risk and for young/less comorbid patients.

**Conclusions:**

Both blood pressure and HbA_1C_ seem to be very important treatment targets, especially in comorbid old patients. It is of particular clinical importance that both over- and under-treatment pose a threat to patients with type 2 diabetes mellitus.

**Electronic supplementary material:**

The online version of this article (doi:10.1186/s12933-015-0179-2) contains supplementary material, which is available to authorized users.

## Background

Amongst the most common chronic diseases, type 2 diabetes mellitus (T2DM) presents some of the greatest clinical and health-economic challenges [1]. In addition to burdens directly associated with this disease, T2DM patients face an increased frequency of micro- and macrovascular complications, hospitalisations and increased mortality rates [2-5].

Treatment of T2DM patients should be based on a broad assessment of patients’ comorbidities, but is, both in scientific discussion as well as in real life care, still mainly focused on tight control of diabetes-related surrogate outcomes such as the body mass index (BMI) or HbA_1C_ [6-12]. However, more recent trials have generated evidence against strict diabetic control (HbA_1C_ <6.5%) with particular relevance for the elderly, mostly comorbid patients as hypoglycemia may represent a particular threat in this age group [13-17]. In further studies, a U-/J-shaped impact of HbA_1C_ on macrovascular event risk was detected indicating that pursuing very low blood glucose levels may be detrimental, at least for specific patient groups [18]. Similar patterns have been reported for another treatment objective, the blood pressure in T2DM patients [19-21]. So, there might be an over- as well as an under-treating risk of T2DM patients with regards to more than one treatment objective.

Most of the existing studies, due to their nature and/or limited sample sizes, do not cover the multimorbid nature of the real-world treatment of T2DM patients. In line with that, studies dealing with the assessment of risk factors possibly associated with diabetes-related events do not differentiate between different outcome types (e.g., micro-/macrovascular events or other event types such as hypoglycaemia) and different patient groups (as defined by age, gender, comorbidities, and/or T2DM severity) [22]. To address these deficits, some authors developed comorbidity-based diabetes complication indices such as the diabetes complications severity index [4] and the adapted diabetes complications severity index (aDCSI) [5]. The available publications show these indexes to correlate with diabetes-related event probabilities and/or all-cause hospitalisations. However, these indexes do not differ between treatment-independent factors which may be influenced by medical treatment (such as demographics or comorbidities) to a lesser degree than treatment-dependent risk factors such as HbA1C, blood pressure, or BMI.

In our study, we had access to a large claims/clinical dataset that included both information on treatment-independent and treatment-dependent factors. Therefore, the aim of this investigation was to analyse which risk factors predict the real-world risk of a macro-/microvascular event, T2DM-related hospitalisation, and all-cause death to happen in T2DM patients. Furthermore, we aimed to investigate whether, with regards to the treatment-dependent factors, there exists both an under- as well as an over-treatment risk.

## Methods

### T2DM sample

We used an anonymised dataset from the German health fund AOK PLUS which included all T2DM patients [at least two T2DM diagnoses as outpatient at independent occasions (ICD E11.-) and/or at least one inpatient T2DM diagnosis in 2010] who were insured by this health fund for the entire study period; death during the observational period was the only exception to the continuous enrolment requirement. The dataset contained information on the sociodemographic characteristics of the patients, their treatment with outpatient medications, their outpatient treatment by GP’s and specialists (diagnoses and frequency of visits), their inpatient treatment in hospitals, and clinical T2DM-related data derived from data documented within a disease management program (DMP) of that health care fund. All patients in this study had to be registered in the T2DM-DMP at the beginning of 2011. As part of the T2DM-DMP, at least once a year and at most quarterly, HbA_1C_, BMI, and blood pressure of each enrolled patient were documented by treating physicians. In summary, our dataset consisted of T2DM patients for which claims data in 2010 and both claims and DMP data in 2011 and 2012 were available.

We analysed the frequency of documented T2DM-related events and/or all-cause death for each study patient. The time period between 01/04/2011-31/12/2012 (7 quarters) was used as observational period, 2010 and the first quarter of 2011 were used as reference period. We included the first quarter of 2011 as reference period because all DMP-based data were only available in 2011 and starting the observation in the second quarter of 2011 ensured that for most patients at least one reference data point regarding HbA_1C_, BMI and blood pressure was available before a potential first event occurred.

### Factors describing treatment-independent disease status of observed T2DM patients

Sociodemographic information and clinical data for each patient were used to describe the disease/comorbidity profile of a patient; these data referred to the reference period. Diabetes-related risk was described based on the aDCSI [4,5]. The aDCSI describes the frequency/severity of observed comorbidities identified by ICD codes from 7 complication categories: retinopathy, nephropathy, neuropathy, cerebrovascular, cardiovascular, peripheral vascular disease, and metabolic complications; it ranges from 0 to 13 and was shown to correlate with the number of all-cause hospitalisations in T2DM patients [4,5] (Additional file 1: Table S1). In order to cover all comorbidities which may not be contained in the aDCSI, we additionally calculated the Charlson Comorbidity index (CCI; Additional file 2: Table S2) [23]. However, because we were going to estimate age as separate risk factor, we excluded age from the CCI (adapted CCI). Because of the relatively short reference period, some comorbidities may not have been documented in our dataset by documented diagnoses which form the basis of the aDCSI and the CCI. So, we additionally calculated the number of different prescribed chronic medications in the reference period (at least two prescriptions per ATC group (4th level, chemical subgroup [24]) in the reference period) as proxy for unobserved comorbidities. Finally, we included in our models occurrence of previous events in 2010 (hospitalisations because of macro-/microvascular events or with T2DM as main diagnosis) as variable describing the morbidity status of the patient.

### Factors describing treatment/treatment efficacy of observed T2DM patients

Treatment and treatment efficacy during the observational period were described based on data on antidiabetic/cardiovascular medication treatment and available HbA_1C_, BMI, and blood pressure values. All variables were included as mean, based on the period from 01/01/2011-31/12/2012. In case that an observed patient experienced at least one of the diabetes-related events as defined further below, the mean of these variables referred to the time period until date of this first event.

The number of outpatient treatment visits (GPs and specialists) was not included as variable describing the treatment/treatment efficacy during the observational period because a higher number of physician visits may indicate a superior treatment, but may also be related to unobserved events or worsening of the disease status of the patients so that this variable would be more a dependent variable than an independent predictor of diabetes-related events and/or death.

Antidiabetic pharmacotherapy was analysed by determining the number of active substances (anatomical therapeutic chemical groups as defined by WHO – ATC groups [24]) prescribed from 01/01/2011 until 31/12/2012 per patient or, in the case of events during the observational period, until an event happened. In order to describe antidiabetic medication patterns (ATC groups A10A and A10B), patients were clustered into the following groups: (a) no antidiabetic therapy, (b) metformin monotherapy, (c) sulfonylureas monotherapy, (d) combination therapy of sulfonylureas/metformin, (e) combination therapy of oral antidiabetics with GLP-1 analogues (f) mono- or combination therapy of DPP-4 inhibitors, (g) combination therapy of OAD/insulin, (h) insulin monotherapy, or (i) other combinations. If different medications were administered sequentially in the observational period, these were described as a combination therapy. Additionally, cardiovascular drug medication therapy was described in the following groups (k) ACE inhibitors, (l) beta blockers, (m) diuretics, (n) statins, and (o) antithrombotic drugs (Vitamin K antagonists and antiplatelets).

### Observed T2DM-related events

Based on previous publications [3,14,18,25], T2DM-related outcomes of our study were considered as follows:

- Macrovascular events
  - Hospitalisations with stroke (ICD 10 I60.-/I61.-/I62.-/I63.-/I64.-)
  - Hospitalisations with acute myocardial infarction (ICD 10 I21.-)
  - Hospitalisations with congestive heart failure (CHF) (ICD 10 I50.-)
  - Hospitalisations with coronary revascularizations (OPS 5-361/5-362/5-363)
  - Hospitalisations with percutaneous transluminal vascular interventions and stent implantations (OPS 8-836/8-837/8-84)
  - Hospitalisations with peripheral vascular disease (ICD 10 I73.9)
  - Hospitalisations with angina pectoris (ICD 10 I20.-)
- Microvascular events
  - Hospitalisations with amputation of the lower extremities (procedural code during hospitalisation: 5-864/5-865)
  - Hospitalisations with vitrectomy (procedural code during hospitalisation: 5-158/5-159)
  - Hospitalisations with chronic kidney disease, stage 5 (ICD 10 N18.5)
- T2DM-related hospitalisations
  - Hospitalisations with T2DM/acute hypoglycaemia as main diagnosis (ICD 10 E11.-/ E16.0/E16.1/E16.2)
  - Death (any cause)
    - Composite outcome consisting of macrovascular/microvascular events, T2DM-related hospitalisations, and all-cause death.

In order to reliably differentiate acute events from previous diagnoses/events, in this analysis ICD diagnoses/procedures documented were only considered as an event if they were either associated with acute hospitalisations as main diagnosis or death of the patient. Main outcome used in this study was a composite outcome (occurrence of any of the above events), in additional analyses the four event types were analysed separately.

### Statistical analysis

The event frequency was reported as event-specific event probability per patient year in 01/04/2011-31/12/2012. Furthermore, percentage of event-free patients over time was depicted by using Kaplan Meier (KM) curves for the whole sample as well as for different patient groups as defined by age, gender, or comorbidity status; significance of differences between event rates was tested by using log-rank tests.

**Figure 1:**
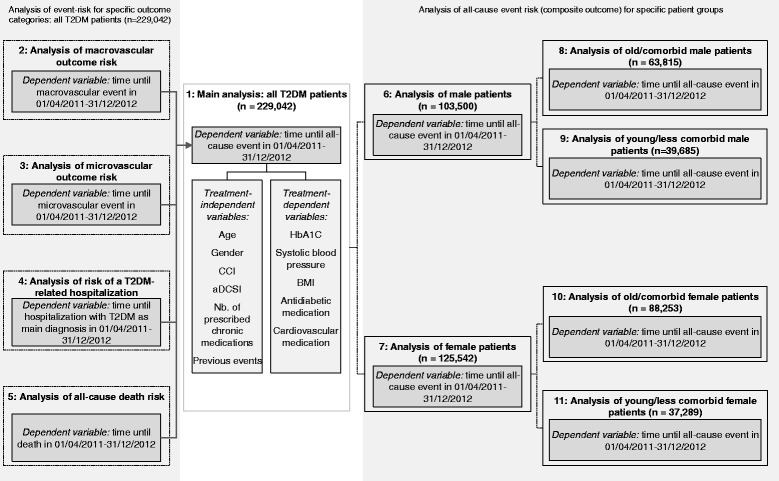
**Overview of different Cox regression models.** The figure describes the 11 different multivariable Cox regression analyses used to assess the association between chosen independent risk factors and observed event risk.

In order to analyse independent factors associated with the observed event risk, we did 11 multivariable Cox regression analyses; Figure 1 describes these models. Factors associated with the time until event in the observational period (01/04/2011-31/12/2012) were analysed using backward elimination methodology. All factors not reaching statistical significance (p < 0.1) were excluded in a stepwise procedure. Finally, factors reaching a p < 0.05 were interpreted as statistically significant. In the models, the following covariates were initially included: as treatment-independent factors age, gender, aDCSI, adjusted CCI as defined above, number of all prescribed chronic medications, occurrence of a previous macro-/microvascular event or a hospitalisation with T2DM as main diagnosis; as treatment-dependent factors (all related to 01/01/2011-31/12/2012 or until time of event, whatever came first): antidiabetic and cardiovascular medication therapy as defined above, mean HbA_1c_, mean BMI, and mean systolic blood pressure. Model 1 analysed all observed T2DM patients estimating time until all-cause event (composite outcome as defined above) as dependent variable. The models 2–5 analysed time until first macrovascular event (model 2), microvascular event (model 3), hospitalisation with T2DM as main diagnosis (model 4), and all-cause death (model 5) separately. In models 6–11, separate patient subgroups as defined by gender and age/comorbidity status were analysed using time until all-cause event (composite outcome) as dependent variable in each of the models. In terms of age/comorbidity status, patients with an age of < 72 years at the beginning of the observational period and an adapted CCI < 4 were interpreted as young/less comorbid patients whereas all other patients were grouped into the older/more comorbid patient subgroups. All reported p-values were two-sided, and 95% CIs were calculated for hazard ratios (HRs). All descriptive evaluations were done with Microsoft SQL Server 2008 and Microsoft Excel 2010. All other statistical analyses were done with SPSS 17.0. The study was approved by the independent ethical review board of the Thuringia State Medical Association (Germany).

## Results

### T2DM sample

In our sample, a total of 229,042 T2DM patients were identified (Table 1). This sample (54.8% female) was characterized by a mean age of 70.2 years and a high number of comorbidities per patient (mean CCI 6.03; mean CCI without age factor: 3.65); the five most common comorbidities were hypertension (86.9% of the patients), lipid metabolism disorders (50.7%), disorders of accommodation/refraction (45.2%), dorsalgia (36.2%) and chronic ischaemic heart disease (34.7%).

**Table 1:** **Sociodemographic characteristics of observed type 2 diabetes mellitus-samples**

**Variables**	**Type 2 diabetes mellitus-prevalent patients with complete DMP-documentation (study sample)**		**Study sample patients which experienced at least one diabetes-related event during the observational period**	
N	229,042		39,589	
Baseline characteristics (reference period)				
Mean age in years (SD)	70.23	(11.12)	74.65	(10.20)
Female gender (%)	125,542	(54.81%)	19,989	(50.49%)
Mean number of long-term prescribed medications (SD) (at least two prescriptions per ATC code - 4th level)	6.10	(3.44)	7.87	(3.76)
Mean CCI (SD)	6.03	(2.83)	7.77	(3.01)
Mean adapted CCI without age factor (SD)	3.65	(2.30)	4.97	(2.67)
Mean aDSCI (SD)	1.92	(1.85)	2.98	(2.09)
Number of patients (%) with at least one previous diabetes-related event	13,967	(6.10%)	5,744	(14.51%)
5 most common comorbidities; Number of patients (%) with				
Hypertension (ICD-10: I10)	199,079	(86.92%)	35,095	(88.65%)
Disorders of lipoprotein metabolism (ICD-10: E78)	116,230	(50.75%)	20,160	(50.92%)
Disorders of refraction and accommodation (ICD-10: H52)	103,471	(45.18%)	16,831	(42.51%)
Dorsalgia (ICD-10: M54)	82,932	(36.21%)	13,327	(33.66%)
Chronic ischaemic heart disease (ICD-10: I25)	79,482	(34.70%)	20,233	(51.11%)
Treatment-dependent variables (based on 01/01/2011 until 31/12/2012 or date of first event)				
Mean HbA_1C_ in % (SD)	7.00	(1.01)	7.24	(1.22)
Number of patients (%) with mean HbA_1C_ <6.0% / <42 mmol/mol	25,445	(11.11%)	3923	(9.91%)
Number of patients (%) with mean HbA_1C_ <7.5% / <58 mmol/mol	172,373	(75.26%)	26,219	(66.23%)
Number of patients (%) with mean HbA_1C_ ≥9.0% / ≥75 mmol/mol	10,308	(4.50%)	3406	(8.60%)
Mean BMI (SD)	30.46	(5.59)	30.07	(5.75)
Number of patients (%) with BMI >30	110,014	(48.03%)	18,003	(45.47%)
Mean systolic blood pressure in mmHg (SD)	135.56	(11.48)	134.33	(13.37)
Number of patients (%) with systolic blood pressure >130 mmHg / >17.3kPa	151,806	(66.28%)	22,789	(57.56%)
Mean diastolic blood pressure in mmHg (SD)	78.75	(6.46)	77.66	(7.48)
Number of patients (%) with diastolic blood pressure >80 mmHg / >10.7kPa	78,511	(34.28%)	10,257	(25.91%)

*Legend:* The table lists sociodemographic characteristics for two different samples: a) type 2 diabetes mellitus-prevalent (2010) patients with complete DMP-documentation (study sample) and b) study sample patients which experienced a diabetes-related event during the observational period.

### Treatment of T2DM patients

A total of 66.3% of our T2DM study sample had a mean systolic blood pressure of > 130 mmHg (mean: 135.56 mmHg). 34.3% of the observed patients had a diastolic blood pressure of > 80 mmHg (mean 78.75 mmHg). 48.0% of the observed patients could be considered as obese (BMI > 30). The mean HbA_1C_ value in the sample was 7.00%; 11.1% of the observed patients had a mean HbA_1C_ < 6.0%, 75.3% of the patients had a mean HbA_1C_ <7.5% and 4.5% of the patients in the study sample had a mean HbA_1C_ ≥ 9.0% (Table 1).

### Diabetes-related events

**Figure 2:**
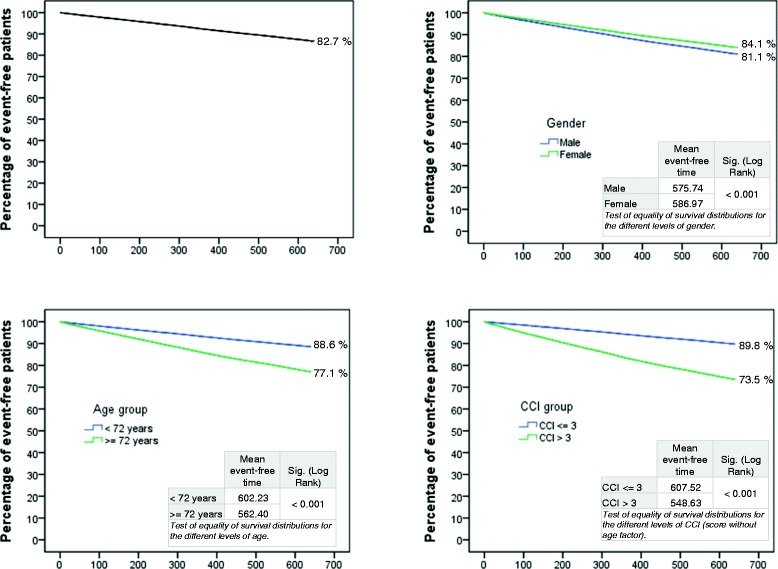
**Kaplan-Meier curves for percentage of event-free patients during observational period.** The figure shows Kaplan-Meier curves regarding the percentage of event-free patients (all-cause event; composite outcome) for the whole sample as well as for different patient groups as defined by age, gender, or comorbidity status.

The mean observational period per patient from 01/04/2011 until 31/12/2012 or until first observed all-cause event was 581.9 days (SD: 148.4). 39,589 patients of the study sample (17.3%) were affected by at least one T2DM-related event in this period. 22,232 patients (9.7%; 82.7 events per 1,000 patient years) were affected by at least one macrovascular event during the observational period, 3,249 patients (1.4%; 10.8 events per 1,000 patient years) suffered from at least one microvascular event, 8,717 patients (3.8%; 28.4 events per 1,000 patient years) experienced at least one hospitalisation with T2DM as main diagnosis, and 15,802 patients (6.9%; 40.7 deaths per 1,000 patient years) died within the observational period. Figure 2 depicts the percentage of event-free patients over time using KM curves. Obviously, event risk was positively associated with older age, male gender, and higher CCI.

### Factors associated with event risk (model 1)

**Figure 3:**
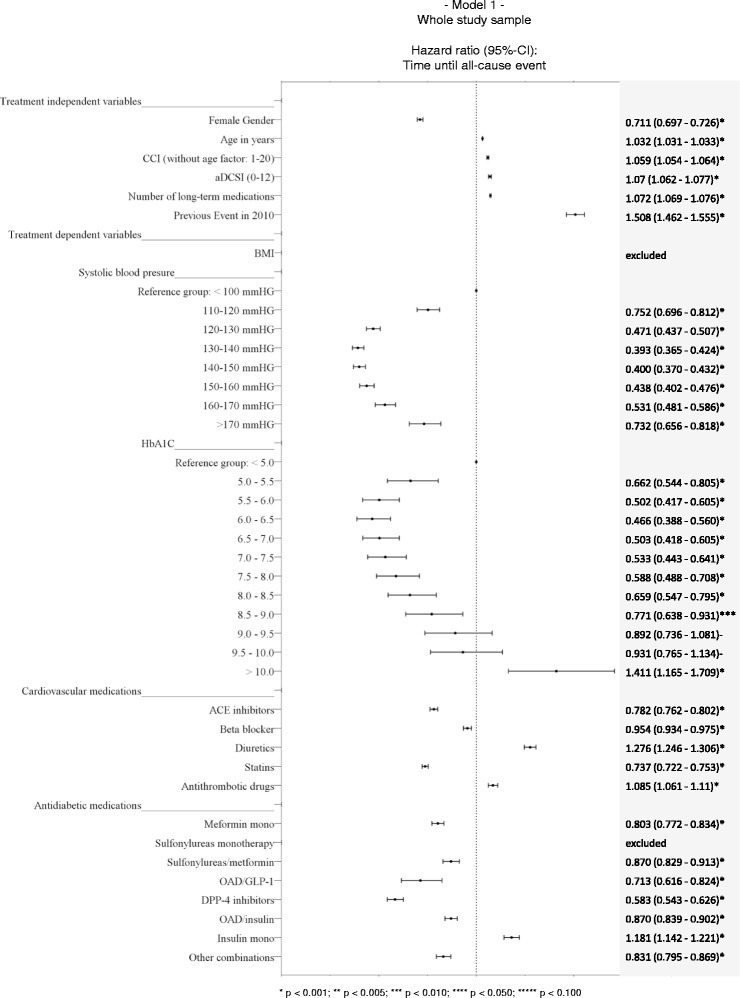
**Factors associated with event risk (model 1).** The figure shows the results of the multivariable analysis with regards to independent factors influencing time until an all-cause event (composite outcome) in the whole study sample.

Figure 3 shows the results of our multivariable analysis regarding factors influencing time until an all-cause event (composite outcome). All included treatment-independent factors did influence the T2DM-related event risk. In our sample, women faced an under-average event risk (HR 0.711) whereas older patients faced a higher event risk (HR 1.032 related to each year of age). The adjusted CCI (HR 1.059 related to values between 1–20), the aDCSI (HR 1.070 related to values between 0–12), the number of prescribed chronic medications (HR 1.072), and any previous event in 2010 (HR 1.508) also positively influenced the event risk.

The mean BMI of the patients was a treatment-dependent factor that was not associated with the T2DM-related event risk in a statistically significant way; so it was excluded based on our backward inclusion methodology. Related to cardiovascular drug therapy, some medications (diuretics: HR 1.276, antithrombotic drugs: HR 1.085) were associated with a higher event risk, others (ACE inhibitors: OR 0.782, statins: HR 0.737, beta blockers HR 0.954) were associated with a lower event risk. In terms of antidiabetic medication therapy, SU monotherapy was excluded from the model because of an insignificant association with event risk. All other observed therapy options except of insulin monotherapy (HR 1.181) were associated with a lower event risk (reference group: no antidiabetic medication).

With regards to systolic blood pressure, we could detect a U-/J-shaped influence on diabetes-related event risk. Using a mean systolic blood pressure of <100 mmHg as reference group, a systolic blood pressure of 130–140 mmHg (HR 0.393) was associated with the lowest event risk; values below/above that “optimal” systolic blood pressure were associated with higher event risks.

The HbA_1C_ as most prominent treatment-dependent risk factor in scientific diabetologic discussion clearly influenced the event risk. We could detect a U-/J-shaped influence of the HbA_1C_ values on diabetes-related event risk. Using a mean HbA_1C_ value of <5% as reference group, a mean HbA_1C_ of 6.0-6.5 was associated with the lowest event risk (HR 0.466). Lower HbA_1C_ values (5.0-5.5: HR 0.662; 5.5-6.0 HR 0.502) seemed to be associated with higher event risks, whereas higher HbA_1C_ values were also associated with a higher event risk; the respective hazard ratios for the HbA_1C_ classes were: 0.503 (6.5-7.0), 0.533 (7.0-7.5), 0.588 (7.5-8.0), 0.659 (8.0-8.5), 0.771 (8.5-9.0), 0.892 (9.0-9.5; not significant), 0.931 (9.5-10.0; not significant), and 1.411 (>10.0).

### Factors associated with specific outcomes risk (models 2–5)

**Figure 4:**
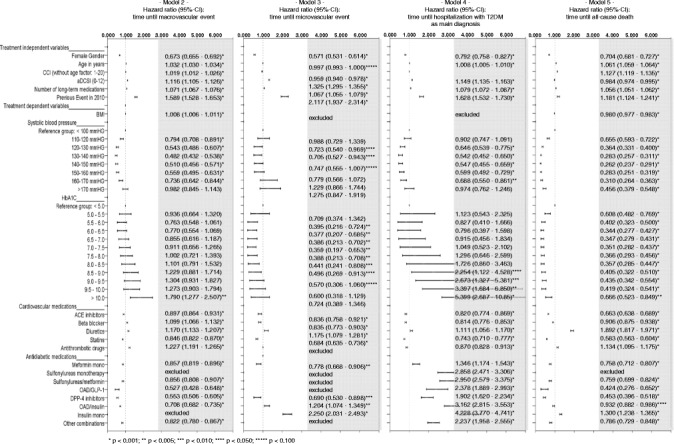
**Factors associated with risk of different events (models 2–5).** The figure compares the results of the multivariable analyses with regards to chosen independent factors being potentially associated with time until a macrovascular event (model 2), a microvascular event (model 3), a diabetes type 2-related hospitalisation (model 4) and all-cause death (model 5) in the whole study sample.

Figure 4 shows results of the multivariable Cox regression estimates with regards to macrovascular event risk (model 2), microvascular event risk (model 3), T2DM-related hospitalisation risk (model 4), and all-cause mortality risk (model 5). As in model 1, female gender, older age (exception: microvascular events), a higher aDCSI, a high number of prescribed medications and any observed previous events are positively associated with the event risk. However, a higher CCI was only associated with a higher macrovascular event and death risk; it did not influence the T2DM-related hospitalisation risk and even moderately decreased the microvascular event risk (HR 0.959).

As described above, BMI as independent factor was excluded from model 1 because of its insignificance. However, a higher BMI was associated with a higher macrovascular event risk (HR 1.008) and a lower death risk (HR 0.980). In terms of cardiovascular medication, the results of model 1 were mainly confirmed in models 2–5. Only results related to beta blockers and antithrombotic drugs differed between the models. Similarly, the results with regards to antidiabetic medication in model 1 could be confirmed in the models 2, 3, and 5; in model 3 (microvascular events), some agents were excluded because of an insignificant influence. However, whereas most of the antidiabetic agents were associated with lower macrovascular/microvascular event and lower death risk, all antidiabetic agents were positively associated with the risk to experience a hospitalisation with T2DM as main diagnosis (model 4). Highest hazard ratios could be detected for insulin monotherapy (HR 4.228), OAD/insulin combination therapy (HR 3.162), sulfonylurea/metformin combination therapy (2.950), and sulfonylurea monotherapy (HR 2.858). 21.2% of the T2DM-related hospitalisations were due to a hypoglycaemia (main diagnosis).

With regards to systolic blood pressure, the observed U/J-curve pattern found in model 1 could only be confirmed for the macrovascular event risk and, even more pronounced, for the mortality risk. Here, a blood pressure of 140–150 mmHg was associated with the lowest death risk (HR 0.262; reference: systolic blood pressure < 100 mmHg), whereas lower and higher blood pressure values were associated with a higher event risk.

With regards to the influence of HbA_1C_ on the event risk, the results also depended on the observed outcome category. The U/J-curve pattern which was found in model 1 could be confirmed in model 5 (death risk). However, in model 2 (macrovascular event risk), only a very high HbA_1C_ > 10% was associated with an elevated event risk. With regards to microvascular complications, a HbA_1C_ value between 5.5-8.0 could be shown as being risk-protective. For T2DM-related hospitalisations, a HbA_1C_ > 8.5 was associated with a higher event risk.

### Factors associated with all-cause event risk in specific patient subsamples (models 6–11)

**Figure 5:**
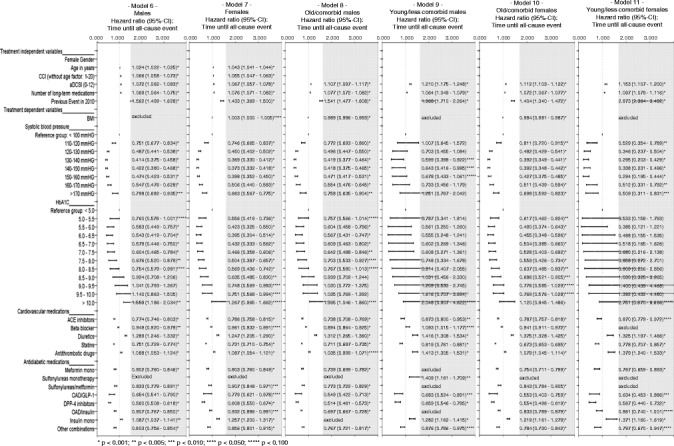
**Factors associated with event risk in different subsample (models 6–11).** The figure compares the results of the multivariable analyses with regards to factors being possibly associated with time until an all-cause event (composite outcome) in the subsamples of male patients (model 6) and female patients (model 7) as well as in the group of old/comorbid male/female patients (models 8/10) and young/less comorbid male/female patients (models 9/11).

Figure 5 shows the results of the multivariable Cox regression estimates with regards to the composite outcome (all-cause events) for 6 patient subgroups: all males (model 6), all females (model 7), old/more comorbid males (model 8), young/less comorbid males (model 9), old/more comorbid females (model 10), and young/less comorbid females (model 11). With regards to sociodemographics/comorbidities, the previously described results could be confirmed across all subgroups. The only specific is the more pronounced influence of previous events in the young/less comorbid patient subgroups. Again, results for BMI as independent risk factor were inconclusive.

Association of certain prescribed cardiovascular drugs with event risk was similar in all models according to the results in model 1. Also, the risk profile associated with specific antidiabetic medication patterns was similar to model 1. However, the risk-increasing association between sulfonylurea monotherapy and all-cause event risk could only be shown in the subsample of young/less comorbid male patients.

The observed U/J curve pattern for the association between systolic blood pressure and all-cause event risk could be confirmed for both male and female patients. However, it seems to be more pronounced in the female patients and old/comorbid patients subsamples. Lowest event risk was associated with a systolic blood pressure (reference group: <100 mmHg) of 130–140 mmHG or 140-150mmHG (old/more comorbid patients).

The observed U/J curve pattern for the association between HbA_1C_ and all-cause event risk could be observed across male and female patients (models 6, 7). The optimal HbA_1C_ of 6.0-6.5 (reference: <5.0) seemed to be more risk-protective for female patients (HR 0.395) than for male patients (HR 0.543). Results of models 9 and 11 show that mean HbA_1C_ was not systematically associated with all-cause event risk in young/less comorbid patients. The observed relationship between HbA_1C_ and all-cause event risk could only be observed in the old/more comorbid patient subsamples (both males/females: models 8 and 10). In these models, a HbA_1C_ of 6.0-6.5 was associated with the lowest event risk.

## Conclusions

### Study objectives and main results

Based on a German claims dataset, we aimed to identify both treatment-independent and treatment-dependent factors that are associated with the T2DM-related event risk. The main strength of our analysis lies in the availability of a very large, data-rich dataset.

Our analysis confirms the influence of age, gender, and number of comorbidities as measured by the adjusted CCI, number of prescribed medications, the aDCSI, and previous events on the event risk as earlier studies did [4,5,25]. In our study, certain medication groups both in terms of cardiovascular and antidiabetic medication were associated with higher/lower event risk rates as well. Because most of these drugs are not substitutes for each other, we do believe that these different event rates are more an indication of existing/undetected comorbidities of patients or T2DM disease severity/length than an indication that treatment of these patients should switch to those drugs that are associated with lower event rates. This is particularly conceivable as a true placebo-control is necessary to validate drug efficacy, but not available here.

Nevertheless, the lower risk of GLP-1, DPP4 or metformin-based treatments versus sulfonylurea and insulin-containing regimens may underpin the advantage of lower rates of hypoglycemia [26] with former ones leading to endpoint-relevant differences in other studies as well [27]. Hypoglycemia is the major downside of sulfonylurea or insulin-based regimens [28] and is one of the major causes of hospitalisations with T2DM as main diagnosis in our study.

In our multivariate statistical analysis, the mean BMI did not exert an independent influence on T2DM-related event rates. Here, our study confirms earlier studies that found it difficult to identify a closely, statistically identifiable relationship between weight/BMI and cardiovascular event rates [28].

Our multivariate statistical analysis shows that treating physicians face a “double-U-/J-curve”-pattern in treating multimorbid T2DM patients. First, we could detect this U-/J-curve shaped influence of systolic blood pressure on diabetic-related events with a systolic blood pressure of 130–140 mmHg as being the optimal range. However, this result is mainly driven by the macrovascular event and death risk. This observation is in line with major T2DM clinical trials such as the UKPDS which clearly showed an even larger endpoint effect of blood pressure than glucose lowering [29]. Also, the observed U-/J-curve pattern was already shown in some studies [19-21,30].

Despite controlling for many covariates, our results support the conclusion that HbA_1C_ remains an important treatment-dependent factor associated with the T2DM-related event risk, even if the analysis controls for different antidiabetic medication therapies. In this, we confirm the results of earlier real life studies [13,14,18,31]. We also confirm that a U-/J-shaped influence of HbA_1C_ on T2DM-related event risk may exist as shown by more recent studies [13,18]. Our data indicate that a HbA_1C_ of 6.0-6.5% may be associated with the lowest T2DM-related event rate and a HbA_1C_ < 6.0% may be associated with higher event rates. This confirms the recently found relationship between hypoglycaemic events and cardiovascular events [32]. However, that does not mean that reduction of HbA_1C_ to a goal of 6.0-6.5% in all (especially older) patients may automatically be associated with an overall reduction of T2DM-related events/mortality as the well-known ACCORD study has shown [33,34]. Furthermore, HbA_1C_ variability has found to be an independent predictor for development of cardiovascular diseases [35].

In terms of specific outcomes, interestingly, our models 2–5 showed that the observed U/J-curve like influence of HbA_1C_ on event risk only exists for the death risk (model 5). For macro-/microvascular events and T2DM-related hospitalisations, we observed the risk-protective nature of low/moderate HbA_1C_ values, but no clear U/J-curve pattern could be shown. Furthermore, models 6–11 demonstrated that HbA_1C_ is hardly related to any outcome risk in young/less comorbid patients. Obviously, death risk is the mainly driver of the close relationship between HbA1C and event risk; mortality risk is generally low in the younger/less comorbid patient groups. Whether mortality risk is, on the other hand, related to macrovascuar causes is not known because no death cause has been documented in the database.

On the other hand, in old/more comorbid patients, influence of HbA1C on overall event risk following a U-/J-curve could be assessed. Here, we cannot confirm the general conclusion that more comorbid T2DM patients may not benefit from low blood glucose values [25,36]; the risk-minimizing HbA_1C_ was a mean value of 6.0-6.5. So, our results support the general conclusion that HbA_1C_ control should be an important part of a general multidisciplinary risk assessment/management effort in an effective T2DM treatment [37].

### Limitations

We acknowledge limitations of our analysis. First, due to the limitations of our dataset we could not include all variables of interest. Patient characteristics not associated with specific diagnoses and/or prescription patterns like smoking/non-smoking behavior, preclinical atherosclerosis [38], specific GFR values [39] or level of physical activity [40] were not visible. As treatment-dependent variable, most importantly, total or LDL-cholesterol values that have been found to be an independent cardiovascular risk factor in other T2DM studies [18,41] were not available. Nevertheless, in our multivariable analysis, statins have been found to be associated with a lower diabetes-related event risk which is in line with clinical findings [42].

Second, less acute events treated in the outpatient sector were not included in our study because differentiation of acute events and earlier events based on outpatient diagnoses only was not possible based on our database. Furthermore, it cannot be ruled out completely that, despite using events related to acute hospitalisations, some of the identified hospitalisations were directly associated with previous events.

Third, despite the fact that we analyzed a very large dataset we covered only a short observational period of 21 months. Further research is needed to describe the more long-term consequences of the analyzed risk factors of T2DM-related events, especially in younger/less comorbid patients with generally lower event rates.

Fourth, our sample consisted of comparatively old/comorbid T2DM patients which led to high average event rates. This is due to the nature of the insured persons of the health care fund AOK Plus which provided the data. So, T2DM patients with higher comorbidity levels are over-represented in our study. Given this, the range for an optimal HbA_1C_ between 6.0 and 6.5 appears to be rather low given those recommendations cited above for elderly and multimorbid patients. However, though representing a relatively old sample, very compromised elderly may not have been included as they did not participate in DMPs and/or were mainly residing in nursing homes. In addition, sicker patients may not have been treated against T2DM at the same intensity as fitter elderly as risk aversion and, thus, reduced treatment intensity had been suggested for those frail patients by former studies. Furthermore, negative experiences (hypoglycemia) prior to the observational period could have resulted in a pre-selection of fitter elderly which were assumed to be less threatened by hypoglycemia than the frail elderly, and a selection of elderly patients with fewer hypoglycemic complications at low HbA1C levels. Despite statistical control, these biases may have resulted in falsely linking tighter T2DM control with favourable outcomes in the elderly. These points could explain the apparent discrepancy bearing in mind that this is no long-term interventional study. In addition, the confidence intervals for the optimal ranges for HbA_1C_ and systolic blood pressure have not been statistically determined, but result from graphical estimation; the true ranges may thus be different from those estimates.

Fifth, we acknowledge that some of the observed/analyzed risk factors may be correlated to each other. This is certainly true for the age, CCI, number of chronic medications and the aDCSI. On the other hand, our analysis aimed to cover comorbid conditions possibly associated with T2DM-related risks as closely and as completely as possible in order to be able to describe the remaining effect of treatment-dependent factors on event risk in the most meaningful way.

Sixth, due to our large sample size, some independent variables may exert a statistical influence but, due to low odds ratios, not in a clinically meaningful way. So, in deriving conclusions, we evaluated both statistical significance and clinical relevance of results.

### Conclusions and implications for practice

Our study confirms that comorbidities play an important role for the prognosis of the micro-/macrovascular event/hospitalisation/death risk in T2DM patients. Both blood pressure and HbA_1C_ seem to be very important targets. It is of particular clinical importance that both over- and under-treatment pose a threat to T2DM patients because both parameters influence diabetes-related event risk and, mainly, mortality risk in a U-/J-shaped pattern.

## Additional files

Additional file 1: Table S1. Components of the aDSCI. The table contains the components of the adapted Diabetes Complications Severity Index and describes the used score methodology based on observed outpatient/inpatient ICD-10 codes in 2010. Additional file 2: Table S2. Charlson-Comorbidity-Index (CCI) and its components. The table outlines the components of the Charlson-Comorbidity-Index (CCI) and describes the used score methodology based on observed outpatient/inpatient ICD-10 codes in 2010.

## Abbreviations

ACCORD: Action to Control Cardiovascular Risk in Diabetes
aDCSI: Adapted Diabetes Complications Severity Index
AD medication: Antidiabetic medication
ATC: Anatomical Therapeutic Chemical
CCI: Charlson Comorbidity Index
CHF: Congestive heart failure
CV medication: Cardiovascular medication
DMP: Disease Management Program
DPP-4: Dipeptidyl peptidase-4
GLP-1: Glucagon-like peptide-1
GP: General practitioner
OAD: Oral antidiabetic drugs
T2DM: Type 2 diabetes mellitus
UKPDS: UK Prospective Diabetes Study
