# Supplementary material for: Treatment-dependent and treatment-independent risk factors associated with the risk of diabetes-related events: a retrospective analysis based on 229,042 patients with type 2 diabetes mellitus
Source: Cardiovasc Diabetol. 2015 Feb 3;14:14. doi: 10.1186/s12933-015-0179-2 (PMC4343042; doi:10.1186/s12933-015-0179-2)
Supplement: Additional file 1: Table S1. — Components of the aDSCI. The table contains the components of the adapted Diabetes Complications Severity Index and describes the used score methodology based on observed outpatient/inpatient ICD-10 codes in 2010. [file 12933_2015_179_MOESM1_ESM.docx]

Table A: Components of the adapted Diabetes Complications Severity Index (aDSCI)

| Complication | Diagnoses | ICD-10 Codes |
| --- | --- | --- |
| Retinopathy (Score 1) | - Diabetic ophthalmologic disease - Background retinopathy - Retinal edema - Other retinal disorders | - E11.3 - H35.0 - H35.8 - H35.9 |
| Retinopathy (Score 2) | - Proliferative retinopathy - Retinal detachment - Blindness - Vitreous hemorrhage | - H35.2 - H33 - H54.0/H54.4 - H43.1 |
| Nephropathy (Score 1) | - Diabetic nephropathy - Acute glomerulonephritis - Nephrotic syndrome - Chronic glomerulonephritis - Nephritis/nephropathy | - E11.2 - N00 - N04 - N03 - N05 |
| Nephropathy (Score 2) | - Renal failure - Renal insufficiency | - N17/N18 - N19 |
| Neuropathy (Score 1) | - Diabetic neuropathy - Amyotrophy - Cranial nerve palsy - Mononeuropathy - Charcot’s arthropathy - Polyneuropathy | - E11.4 - G73.0 - H49.0/H49.1/H49.2 - G59.0 - M14.6 - G63.2 |
| Cerebrovascular (Score 1) | - TIA | - G45.9 |
| Cerebrovascular (Score 2) | - Stroke | - I63/I64/I69 |
| Cardiovascular (Score 1) | - Atherosclerosis - Other IHD - Angina pectoris - Other chronic IHD | - I70 - I23/I24 - I20 - I25 |
| Cardiovascular (Score 2) | - Myocardial infarction - Ventricular fibrillation, arrest - Atrial fibrillation, arrest - Old myocardial infarction - Heart failure - Aortic aneurysm/dissection | - I21 - I49.0 - I48 - I22 - I50 - I71/I72 |
| Peripheral vascular disease (Score 1) | - Diabetic PVD - PVD/Claudication, intermittent - Foot wound + complication | - E11.5 - I73.9 - S91 |
| Peripheral vascular disease (Score 2) | - Embolism/thrombosis (LE) - Gangrene - Gas gangrene - Ulcer of lower limbs | - I74.3 - R02 - A48 - L97 |
| Metabolic (Score 2) | - Ketoacidosis - Other coma | - E11.1 - E11.0 |
