# Supplementary material for: Treatment-dependent and treatment-independent risk factors associated with the risk of diabetes-related events: a retrospective analysis based on 229,042 patients with type 2 diabetes mellitus
Source: Cardiovasc Diabetol. 2015 Feb 3;14:14. doi: 10.1186/s12933-015-0179-2 (PMC4343042; doi:10.1186/s12933-015-0179-2)
Supplement: Additional file 2: Table S2. — Charlson-Comorbidity-Index (CCI) and its components. The table outlines the components of the Charlson-Comorbidity-Index (CCI) and describes the used score methodology based on observed outpatient/inpatient ICD-10 codes in 2010. [file 12933_2015_179_MOESM2_ESM.pptx]

## Slide 1
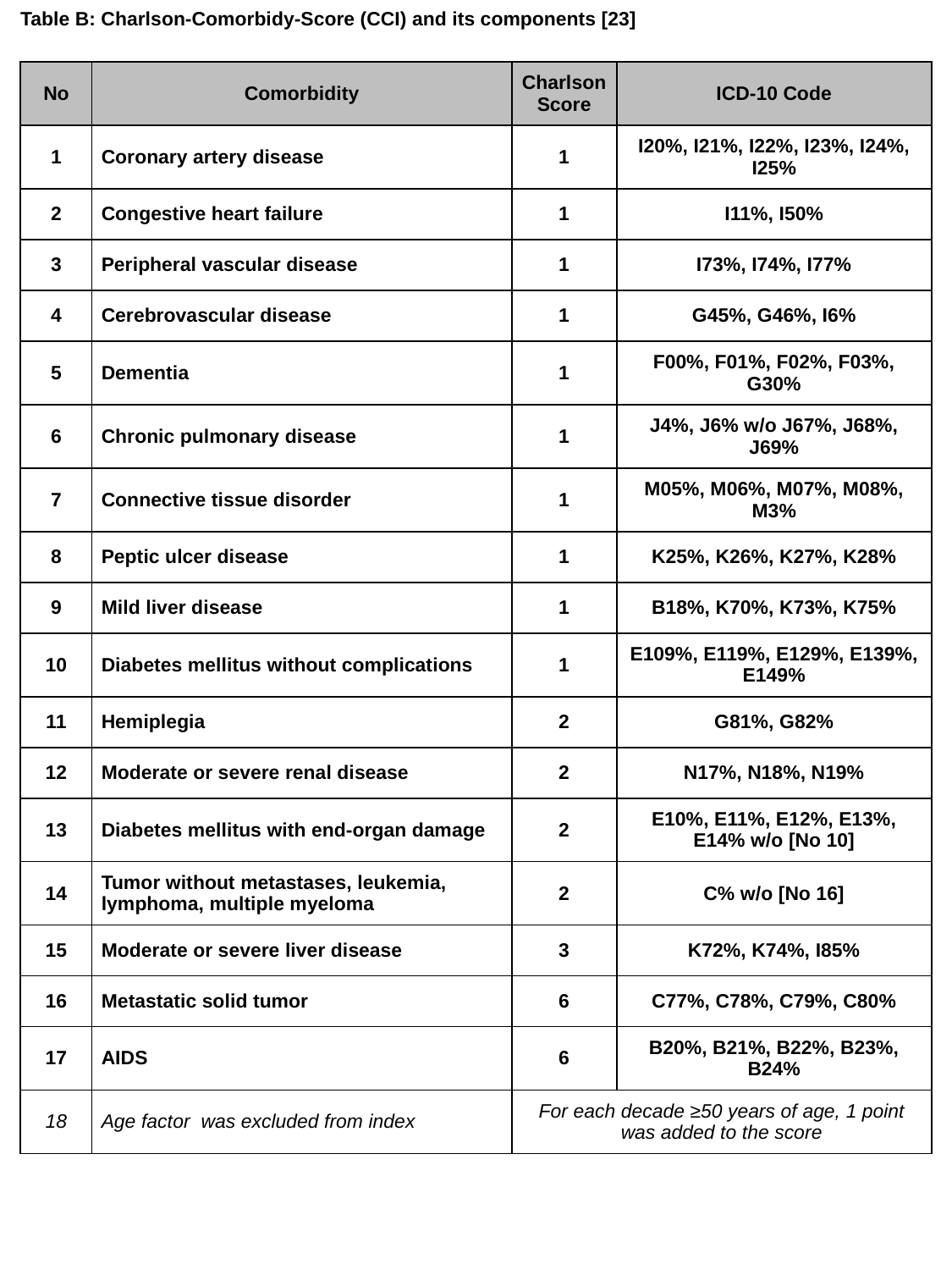

Table B: Charlson-Comorbidy-Score (CCI) and its components [23]
| No | Comorbidity | Charlson Score | ICD-10 Code |
| --- | --- | --- | --- |
| 1 | Coronary artery disease | 1 | I20%, I21%, I22%, I23%, I24%, I25% |
| 2 | Congestive heart failure | 1 | I11%, I50% |
| 3 | Peripheral vascular disease | 1 | I73%, I74%, I77% |
| 4 | Cerebrovascular disease | 1 | G45%, G46%, I6% |
| 5 | Dementia | 1 | F00%, F01%, F02%, F03%, G30% |
| 6 | Chronic pulmonary disease | 1 | J4%, J6% w/o J67%, J68%, J69% |
| 7 | Connective tissue disorder | 1 | M05%, M06%, M07%, M08%, M3% |
| 8 | Peptic ulcer disease | 1 | K25%, K26%, K27%, K28% |
| 9 | Mild liver disease | 1 | B18%, K70%, K73%, K75% |
| 10 | Diabetes mellitus without complications | 1 | E109%, E119%, E129%, E139%, E149% |
| 11 | Hemiplegia | 2 | G81%, G82% |
| 12 | Moderate or severe renal disease | 2 | N17%, N18%, N19% |
| 13 | Diabetes mellitus with end-organ damage | 2 | E10%, E11%, E12%, E13%, E14% w/o [No 10] |
| 14 | Tumor without metastases, leukemia, lymphoma, multiple myeloma | 2 | C% w/o [No 16] |
| 15 | Moderate or severe liver disease | 3 | K72%, K74%, I85% |
| 16 | Metastatic solid tumor | 6 | C77%, C78%, C79%, C80% |
| 17 | AIDS | 6 | B20%, B21%, B22%, B23%, B24% |
| 18 | Age factor was excluded from index | For each decade ≥50 years of age, 1 point was added to the score | |
